# Disruption of medial prefrontal synchrony in the subchronic phencyclidine model of schizophrenia in rats

**DOI:** 10.1016/j.neuroscience.2014.12.014

**Published:** 2015-02-26

**Authors:** A.M.J. Young, C. Stubbendorff, M. Valencia, T.V. Gerdjikov

**Affiliations:** a School of Psychology, College of Medicine, Biological Sciences and Psychology, University of Leicester, United Kingdom; b Neurophysiology Laboratory, Neuroscience Area, CIMA, Universidad de Navarra, Pamplona, Spain

**Keywords:** schizophrenia, phencyclidine, PCP, neurophysiology, medial prefrontal cortex, theta oscillations, LFP, local field potential, MK-801, dizocilpine, mPFC, medial prefrontal cortex, NMDA, N-methyl-d-aspartate, PCP, phencyclidine, PFC, prefrontal cortex

## Abstract

Subchronic treatment with the N-methyl-d-aspartate (NMDA) antagonist phencyclidine (PCP) produces behavioral abnormalities in rodents which are considered a reliable pharmacological model of neurocognitive deficits in schizophrenia. Alterations in prefrontal neuronal firing after acute PCP administration have been observed, however enduring changes in prefrontal activity after subchronic PCP treatment have not been studied. To address this we have recorded cortical oscillations and unit responses in putative cortical pyramidal cells in subchronic PCP-treated rats (2 mg/kg twice daily for 7 days) under urethane anesthesia. We found that this regimen reduced theta oscillations in the medial prefrontal cortex. It further produced abnormal cortical synchronization in putative cortical pyramidal cells. These alterations in prefrontal cortex functioning may contribute to cognitive deficits seen in subchronic NMDA antagonist pre-treated animals in prefrontal-dependent tasks.

## Highlights

• Subchronic PCP pretreatment reduced theta oscillations in medial prefrontal cortex.
• Subchronic PCP pretreatment produced abnormal cortical synchronization in putative cortical pyramidal cells.
• Subchronic PCP pretreatment produced abnormal locking of cortical spikes to lower oscillation frequencies.

## Introduction

Subchronic treatment with the non-competitive N-methyl-d-aspartate (NMDA) receptor antagonist phencyclidine (PCP) in rodents is used to model cognitive deficits in schizophrenia. PCP-treated rats exhibit impaired performance on a number of behavioral tests thought to parallel cognitive and negative symptoms of human schizophrenia (Javitt et al., 2012; Neill et al., 2014). Deficits include changes in prepulse inhibition, attention, working memory and set shifting (Egerton et al., 2008; Neill et al., 2010). The mechanisms through which PCP produces behavioral impairments is largely unknown, however neurophysiological and immunohistochemical results point to the involvement of prefrontal cortical circuits (Li et al., 2010; Kargieman et al., 2012; Celada et al., 2013). NMDA antagonist models of schizophrenia predict deficits correlated with NMDA receptor distribution. Thus NMDA receptors are present at high density in frontoparietal and temporal brain regions suggesting that disruptions in this system underlie cognitive deficits associated with this condition (Javitt et al., 2012). Impaired prefrontal cortex (PFC) function in acute NMDA antagonist-treated rats parallels findings implicating PFC in the neuropathology of schizophrenia. Prefrontal anatomical, cellular and neurochemical alterations have been reported in schizophrenic patients (Rajkowska et al., 1998; Cullen et al., 2006; Ellison-Wright and Bullmore, 2010).

There is robust evidence that acute NMDA receptor blockade alters prefrontal neurophysiological function *in vivo*. In rats, acute systemic PCP administration exerts a complex effect on the discharge rate of pyramidal neurons in medial prefrontal cortex (mPFC), resulting primarily in excitation but also in inhibition in some units, as well as altered firing patterns and locking to ongoing slow oscillations (Kargieman et al., 2007, 2012). The overall excitatory effects of acutely administered NMDA receptor antagonists on prefrontal activity may be mediated by suppression of GABAergic activity and likely results from network interactions which may involve thalamic, basal ganglia and hippocampal regions. Acute NMDA receptor blockade promotes hyperconnectivity in functional brain networks (Dawson et al., 2014a). This maps onto findings that positive symptomatology in human patients is associated with prefrontal hyperactivity (Shergill et al., 2000). However a distinction must be made between schizophrenia-like deficits induced with acute vs. subchronic NMDA-receptor blockade. Thus repeated subchronic PCP exposure produces long-lasting behavioral deficits, which far outlast the period of drug infusion (Neill et al., 2010). It also results in sustained NMDA receptor hypoactivity and disruptions in prefrontal metabolism, reduced expression of the GABA marker parvalbumin, and compromised functional integration between distributed neural systems assessed with cerebral glucose utilization (Dawson et al., 2014b).

Prefrontal neurophysiological parameters have not been characterized *in vivo* after subchronic NMDA receptor blockade. To address this here we recorded activity in putative pyramidal neurons in mPFC of drug-free rats with a history of repeated PCP dosing. This ensured that the reported deficits reflected enduring, conformational changes brought about by PCP pretreatment rather than acute drug effects. We found that subchronic PCP disrupted low-frequency oscillations in mPFC and resulted in abnormal cortical synchrony in this structure.

## Experimental procedures

### Subjects

Male Wistar rats, obtained from Charles River (Margate, Kent, UK), weighing between 200 and 250 g on arrival were housed in pairs on a 12-h reversed light–dark cycle (lights on at 1900 h) at an average temperature of 21 °C and humidity of 40–70%. Water and food (LabDiet 5LF5, PMI Nutrition Intl, Brentwood, MO, USA) were freely available. The experiments were carried out under institutional ethics approval and appropriate project and personal license authority granted by the UK Home Office under the Animals (Scientific Procedures) Act 1986.

### PCP treatment

One week after arrival, rats received pre-treatment of PCP hydrochloride (Sigma Aldrich, Gillingham, Dorset, UK; product nr. P3029) (2.0 mg/kg; *n* = 7) or saline (*n* = 8), in a volume of 1 mL/kg i.p. twice daily for 7 days. Following PCP and saline treatment, the animals were given a 1-week drug-free period prior to testing. The PCP dosing regimen was based on previous work by us and others demonstrating robust deficits in exploratory and attentional paradigms and neurochemical deficits (McLean et al., 2008; Sood et al., 2011).

### Electrophysiological recordings and analysis

Acute electrophysiological recordings were performed under urethane anesthesia (1.5-mg/kg i.p.). Body temperature was monitored rectally and maintained at 37 °C using a homeothermic pad (Harvard Apparatus, Boston, MA, USA). For fluid replacement, 5% glucose was continuously administered via an infusion pump (3 mL/h, s.c.; Instech, K. D. Scientific, Holliston, MA, USA). Glycopyrronium bromide (40 μL/kg, i.m.; Anpharm, Warsaw, Poland) was given to reduce respiratory tract secretions. Animals were fixed to a stereotaxic frame and the head was adjusted so that lambda and bregma were on the same horizontal plane. To prevent corneal desiccation Lacri-Lube Eye Ointment (Allergan, Wesport, Ireland) was applied to the eyes.

mPFC was targeted with a left-side craniotomy with coordinates: +3.2 mm AP; 0.5 mm ML; −2 mm DV (Paxinos and Watson, 2007). Recording electrodes consisted of quartz glass-coated platinum/tungsten wires pulled and ground to custom shapes in our laboratory (shank diameter 80 μm; diameter of the metal core 23 μm; free tip length ∼8 μm; impedance, 1–3 MΩ; Thomas Recording, Giessen, Germany). Wideband signals were acquired continuously via an op-amp-based headstage amplifier (HST/8o50-G1-GR, 1x gain, Plexon Inc., Dallas, TX, USA), passed through a preamplifier (PBX2/16wb, 1000x gain; Plexon Inc., Dallas, TX, USA) and digitized at 40 kHz. For spike sorting the raw signal was band-pass filtered 300–3000 Hz and spikes were sorted using the Matlab-based Wave_clus software to yield single-unit spike trains (Quiroga et al., 2004). Wave_clus performs unsupervised spike detection and sorting using wavelets and super-paramagnetic clustering. All automatic detection thresholds and sorting solutions were examined individually and adjusted if needed. Field potentials were low-pass filtered using a 200-Hz cut-off Butterworth filter and downsampled offline to 5000 Hz. Power spectral densities of the signals recorded from PFC were estimated by means of Welch periodograms (window length: 2 s, overlap: 90%, Hanning window, resolution 1 Hz/bin). Treatment effects in the energy values (mean power of all frequencies within the band) for the delta (0.5–3.5 Hz), theta (4–7.5 Hz), alpha (8–12 Hz), mu (10–12 Hz), beta (13–30 Hz) and gamma (30–70 Hz) frequency bands were assessed using the Mann–Whitney *U* test for equal medians. To investigate drug effects on spike-local field potential (LFP) synchrony, the LFP signal was band-pass filtered using a 3-Hz moving window with a second-order Butterworth filter. A Hilbert transform was applied to obtain the instantaneous phase for each frequency range (Fig. 3A, B) (Saleem et al., 2010). Average phase angle for each spike at each frequency window was calculated using the Matlab toolbox CircStat (Berens, 2009). Further analyses were calculated using Neuroexplorer (Nex Technologies, Littleton, MA, USA) and custom-written Matlab routines.

## Results

### mPFC theta spectral power is reduced by PCP pretreatment

**Fig. 1:**
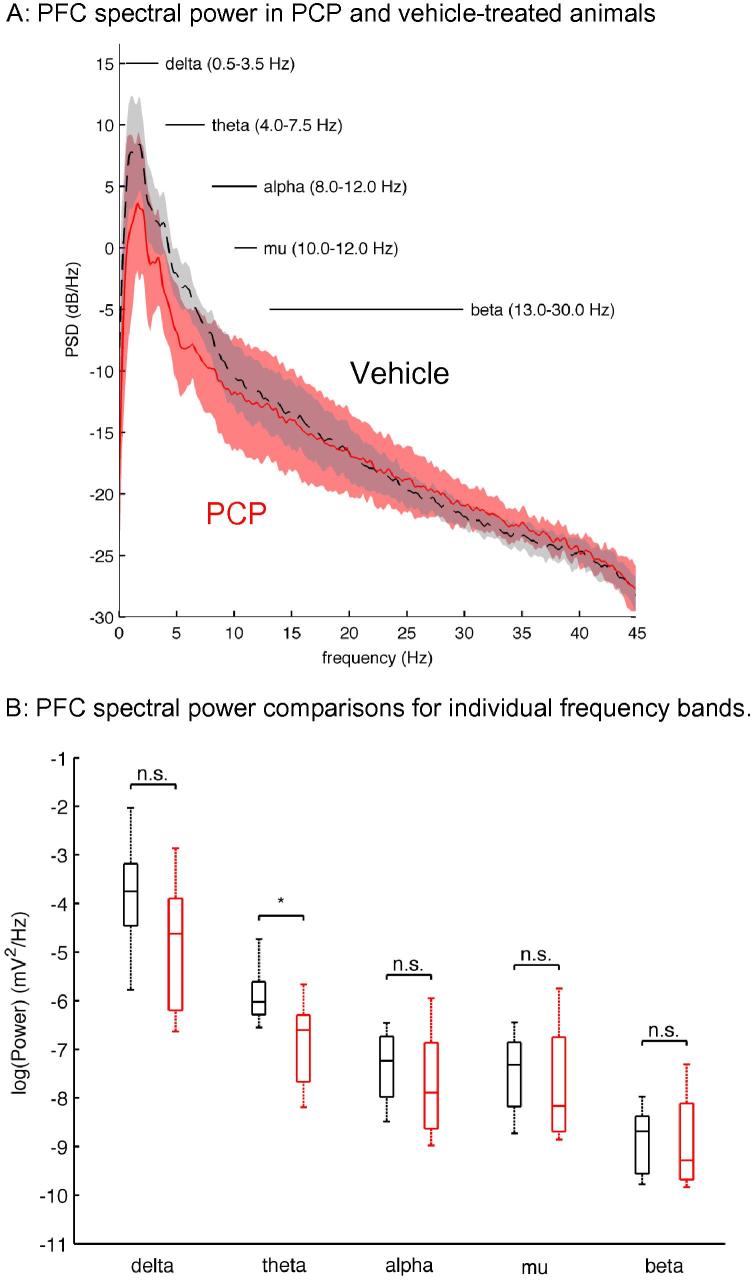
Power spectral density (PSD) of mPFC oscillatory activity shows a significant decrease in theta power in PCP-treated animals. (A) Power spectral density of mPFC oscillatory activity in vehicle- and PCP-treated animals. Data are depicted as mean ± CI, gray traces for vehicle, red for PCP-treated animals. (B) Box-plot representation for the energy at each of the frequency bands of interest for the vehicle (black boxes) and PCP-treated conditions (red boxes). Statistical tests (Mann–Whitney *U* test) reveal a significant decrease (*p *= 0.04) in the energy of the theta band (4–7.5 Hz). (For interpretation of the references to colour in this figure legend, the reader is referred to the web version of this article.)

Spectral analysis of LFP activity from mPFC of vehicle- or PCP-treated rats showed that the predominant activity in both groups was gathered in the lower frequency ranges (Fig. 1A). Qualitatively, PCP-treated rats showed a decrease in the energy of the oscillatory activity at the lower frequency bands (Fig. 1B). Significant statistical differences emerged for the theta range (Mann–Whitney *U* test, *p *= 0.0 4). For higher frequencies, the spectra of both groups decayed monotonically and no prominent activity was detected.

### PCP pretreatment results in abnormal cortical synchrony

**Fig. 2:**
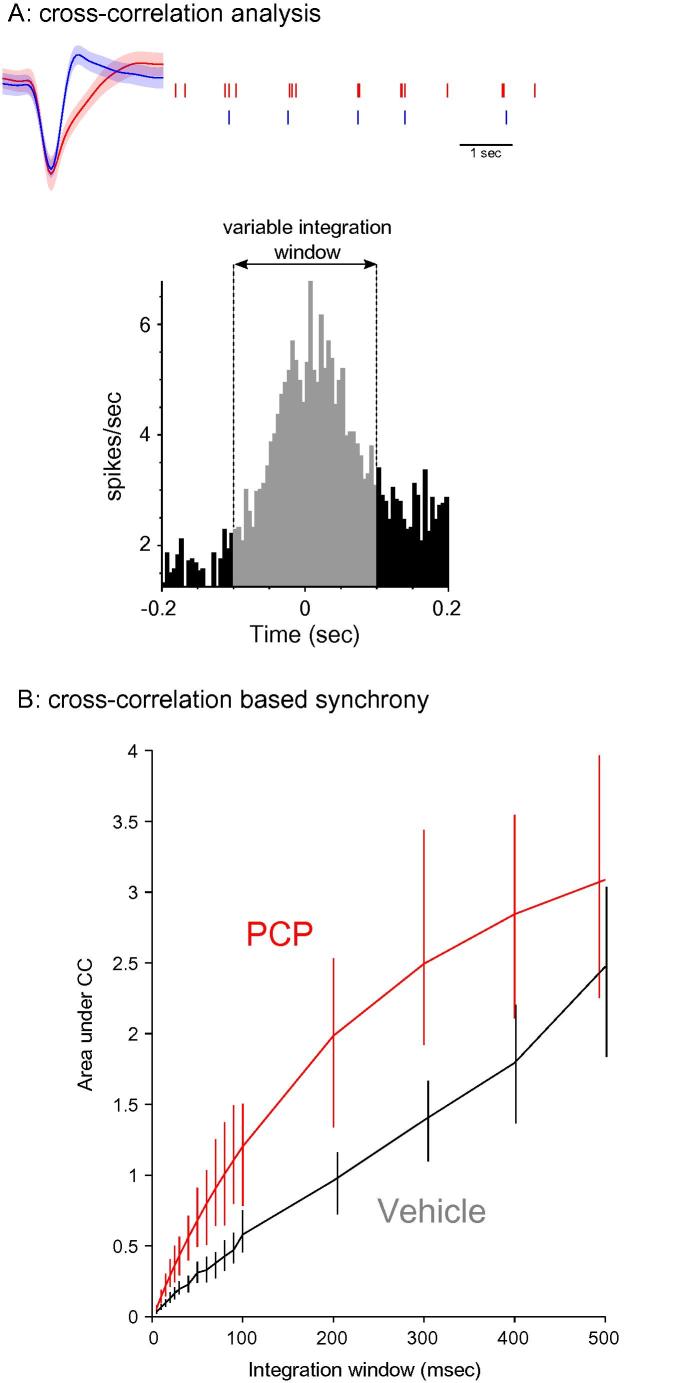
Spike cross-correlations reveal abnormal prefrontal synchrony in PCP-treated animals. (A) Illustration of the cross-correlation-based analysis from two mPFC units recorded simultaneously; area under the cross-correlation was integrated over windows of varying length and plotted in (B). Spike waveform error bands represent SD. (B) Cross-correlogram-based synchrony was significantly higher in PCP-treated animals for a range of integration windows. Error bars represent a 95% bootstrapped confidence interval**.**

The reduction in theta power suggested deficits in mPFC synchrony. To further investigate this observation we analyzed synchrony looking at both single-unit activity and spike-LFP locking. We analyzed 29 units in PCP-treated animals and 48 units in drug-naïve controls. In order to target putative pyramidal cells we recorded from units with relatively low firing rates. Firing rates did not differ significantly across the two groups: 0.90 Hz for saline (SEM = 0.48) and 1.12 for PCP (SEM = 0.51) and are consistent with the cells being regularly spiking units. To confirm this we calculated waveform shapes and these are largely consistent with previous work [e.g. (Bruno and Simons, 2002); initial wave duration (mean ± sd) was 0.64 ± 0.3036 ms and the second phase (corresponding to spike after-hyperpolarization) was 0.87 ± 0.34 ms]. To investigate spike synchrony we computed the cross-correlogram of spikes recorded simultaneously and calculated the area under the cross-correlogram integrating over windows of varying length (Fig. 2A). The area under the cross correlogram was significantly higher (95% bootstrapped confidence intervals) in PCP pre-treated animals over a range of window durations suggesting increased synchrony (Fig. 2B). To rule out the possibility that cluster cutting may influence this conclusion we calculated average auto-correlograms for PCP and vehicle-pretreated groups; the autocorrelograms did not differ significantly between conditions. During slow wave sleep, quiet wakefulness, and some forms of anesthesia including urethane, brain states are characterized by low-frequency, large-amplitude membrane potential changes (Steriade et al., 1993; Petersen et al., 2003). Here we found no difference in overall slow oscillation activity (<3.5 Hz) as a result of PCP treatment (Fig. 1). Still to rule out the possibility that spike cross correlations may be differentially affected by these so-called UP and DOWN states in the drugs vs. vehicle group we calculated the instantaneous oscillation phase of each spike using a Hilbert transformation of the low-pass (<3 Hz) filtered LFP trace to find spike occurrences during UP and DOWN states. Integrated averages of cross-correlations calculated from spike pairs occurring in either UP or DOWN states produced similar drug differences. Therefore we conclude that urethane-produced slow oscillations are unlikely to account for enhanced spike synchrony in PCP-treated animals.

**Fig. 3:**
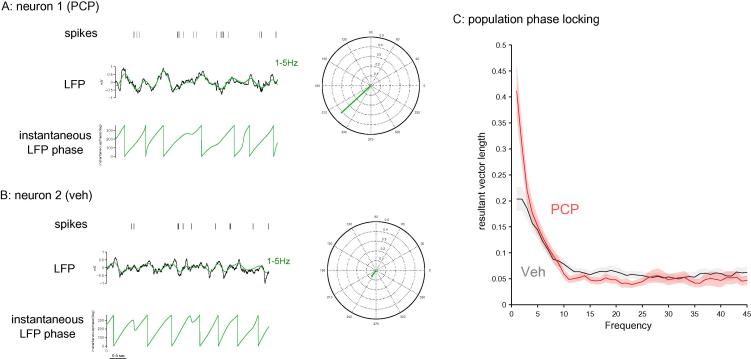
mPFC putative pyramidal neurons show enhanced synchrony with lower LFP frequencies in PCP-treated animals. (A) Example of single-unit activity in a neuron recorded from a PCP-treated animal (top) juxtaposed to ongoing LFP oscillations (middle) recorded from the same electrode and filtered at 1–5 Hz (green trace). The instantaneous phase (bottom) of the 1–5-Hz oscillations is obtained using a Hilbert transformation. It was used to calculate resultant vector length for spike times relative to the phases of the 1–5-Hz frequency band as shown in the polar plot on the right. Resultant vector length reflects the magnitude of the directionality effect and thus represents the degree of locking. (B) Example of single-unit activity juxtaposed to ongoing LFP oscillations in a neuron recorded from a saline-treated animal; data are represented as in (A). (C) Population locking of single units across frequencies for PCP and saline-treated animals. Neurons recorded in PCP-treated animals showed enhanced locking to lower frequencies. Error bands are calculated across neurons and represent SEM. (For interpretation of the references to colour in this figure legend, the reader is referred to the web version of this article.)

Next we investigated mPFC abnormal synchronization in PCP pre-treated animals using another, independent metric based on spike-LFP locking. We calculated mean resultant vector length of each single unit relative to discrete LFP frequency components obtained by sliding a 3-Hz-wide window along the frequency domain (Fig. 3A, B). Resultant vector length reflects the magnitude of the directionality effect and thus represents the degree of locking. Population averages clearly revealed higher locking to lower frequencies in PCP pre-treated animals (Fig. 3C). This finding cannot be explained with group differences in urethane-induced slow oscillations because delta spectral power was not affected by PCP pre-treatment (see Fig. 1B). These results point to PCP-induced abnormal synchronization in PFC. Taken together with observations of reduced theta-band activity they may represent a compensatory mechanism which in behaving animals could mask dynamic, task-related changes in single-unit synchrony and produce cognitive deficits in PFC-dependent tasks (Benchenane et al., 2010; van Wingerden et al., 2010).

## Discussion

Subchronic PCP treatment in rodents produces a number of behavioral abnormalities which model cognitive and negative symptoms of schizophrenia. Here we studied for the first time prefrontal neurophysiological deficits in this pharmacological model. We found that subchronic PCP produced distinct prefrontal deficits including disrupted prefrontal theta power and abnormal synchronization in pyramidal cells. Consistent with the role of prefrontal synchrony in attention, these deficits may drive cognitive abnormalities observed after subchronic NMDA antagonist treatment.

Intrinsic oscillatory activity in the cortex underlies the modulation, conditioning and redirection of incoming information (Benchenane et al., 2010). In the current study LFP spectral analyses revealed a decrease in mPFC theta power, a frequency range involved in attention and working memory. Consistent with this observation, behavioral demands imposed by a spatial working memory task modulate mPFC theta and subchronic NMDA antagonist-pretreated mice are impaired in spatial working memory (Mandillo et al., 2003). PCP effects on theta power may suggest dysregulation of local inhibitory circuits which is consistent with immunochemical studies in PCP-treated rats and post-mortem schizophrenic brains (Beasley et al., 2002; McKibben et al., 2010). Work in the hippocampus shows that theta oscillations are modulated by GABA transmission and that firing of parvalbumin-positive GABA neurons is strongly coupled to theta (Klausberger and Somogyi, 2008; Yu et al., 2013). GABA transporter 1 knockout mice show reduced hippocampal theta and behavioral abnormalities related to schizophrenic symptoms which may be specific to prefrontal GABA transmission (Gong et al., 2009). At present it is not clear whether the disruption of theta activity observed here is secondary to alterations in hippocampal theta. Thus *Df(16)A^+/−^* mice, a model of a recurrent genomic disorder with strong links to schizophrenia and cognitive dysfunction, show disrupted prefrontal-hippocampus LFP coherence (Sigurdsson et al., 2010). Prefrontal theta power was not altered in *Df(16)A^+/−^* mice and behavioral abnormalities in this mouse line are different from the one observed in the chronic PCP model: the authors report spontaneous hyperactivity whereas hyperlocomotion is not seen in repeated NMDA-antagonist-treated rodents tested drug-free (Stark et al., 2008). However while some but not all rodent models show disrupted prefrontal hippocampal coherence, recent work suggests that mPFC theta does not depend on hippocampal input as inactivation of the ventral hippocampus had no effect on mPFC theta (O’Neill et al., 2013). Notably, these models are in contrast with EEG measurements in schizophrenic patients which show increased power in the lower frequency bands including theta although decreases in delta power during sleep have also been reported (Keshavan et al., 1998; Sekimoto et al., 2007; Narayanan et al., 2014). The use of anesthesia in the current study, the global nature of the EEG readout and medication effects in patients may contribute to these differences; further work is clearly required to better map these models to neuropsychiatric variables.

Here we also report abnormal synchrony in mPFC of PCP-treated animals. Pyramidal cells showed stronger cross-correlations and abnormally high locking to lower frequency bands of ongoing LFP oscillations. On the other hand acute MK-801 treatment results in reduction of mPFC firing synchrony (Molina et al., 2014). Thus our finding appears specific to negative symptomatology with which the subchronic PCP model is most strongly associated and is consistent with *in vitro* observations of hypersensitive responses of mPFC neurons to NMDA and depolarizing currents (Arvanov and Wang, 1999; Ninan et al., 2003). Cognitive tasks in drug-naïve rodents may offer clues about the functional significance of the disruptions observed in the present study. Thus orbitofrontal single units lock to theta-band oscillations during reward expectation and current source density analysis suggested a local source of the theta oscillation (van Wingerden et al., 2010). Reward expectation also enhanced theta power in this study. Increased baseline locking as reported in the current study may mask phasic synchrony-based signals driving attentional deficits in this model.

The mechanisms of acute vs. sub-chronic PCP treatment are likely to be different. Acute PCP results in robust increases in locomotor activity whereas overall baseline activity was not increased after repeated NMDA receptor antagonist dosing in rats or mice [(Mandillo et al., 2003; Schlumberger et al., 2009; Beninger et al., 2009); but see also (Jentsch et al., 1998)]. Acute NMDA-antagonist treatment may recapitulate the acute psychotic symptoms of the disorder whereas chronic NMDA antagonist treatments may be more closely related to cognitive deficits and negative symptoms (Dawson et al., 2014b). Patients in the prodromal and first-episode phases of schizophrenia show enhanced fronto-temporal BOLD connectivity during a working memory task compared to negative coupling in controls (Crossley et al., 2009). Consistent with this, recent fMRI work shows increased connectivity within PFC in rats after acute ketamine administration (Gass et al., 2014). On the other hand, subchronic PCP produces compromised functional integration including between hippocampus and PFC (Dawson et al., 2014b). Thus the deficits we report here are likely to be due to relatively permanent synaptic adaptations in PFC resulting from repeated PCP-produced activation (Elsworth et al., 2011). Acute systemic PCP or MK801 administration produces tonic excitation of PFC neurons and increases dopamine efflux and these effects may be mediated by hippocampal input to PFC rather than local effects of the drug (Blot et al., 2013; Moghaddam and Adams, 1998; Jodo, 2013). On the other hand repeated administration produces structural changes in mPFC synapses suggesting that neurophysiological deficits have a local origin. Our results reinforce the hypothesis that schizophrenia-like symptomatology seen in rats after sub-chronic PCP treatment is different from deficits produced by acute PCP. Thus in parallel with results in increased locomotion in rodents after acute NMDA antagonist administration, in humans deficits produced by acute ketamine administration correlate with levels of prefrontal dopamine release (Aalto et al., 2005). On the other hand, the decreases in theta power observed in the current study are likely to be unrelated to prefrontal dopaminergic transmission because mPFC iontophoretic application of dopamine has no effects on prefrontal theta power (Benchenane et al., 2010). This is consistent with evidence for non-dopaminergic mechanisms of PCP-induced cognitive abnormalities (Carlsson and Carlsson, 1989; Koek et al., 1989). This study represents the first analysis of prefrontal neurophysiological sequela of subchronic NMDA antagonist administration in rats. We found that repeated PCP treatment disrupts mPFC theta oscillations. It further produced abnormal cortical synchronization in pyramidal cells. Consistent with the role of prefrontal synchrony in attention, these neurophysiological deficits may drive widely reported cognitive abnormalities after subchronic NMDA antagonist treatment.
